# Differential cerebral response to somatosensory stimulation of an acupuncture point vs. two non-acupuncture points measured with EEG and fMRI

**DOI:** 10.3389/fnhum.2015.00074

**Published:** 2015-02-13

**Authors:** Till Nierhaus, Daniel Pach, Wenjing Huang, Xiangyu Long, Vitaly Napadow, Stephanie Roll, Fanrong Liang, Burkhard Pleger, Arno Villringer, Claudia M. Witt

**Affiliations:** ^1^Mind-Brain Institute at Berlin School of Mind and Brain, Charité – Universitätsmedizin Berlin and Humboldt-UniversityBerlin, Germany; ^2^Department of Neurology, Max Planck Institute for Human Cognitive and Brain SciencesLeipzig, Germany; ^3^Institute for Social Medicine, Epidemiology, and Health Economics, Charité – Universitätsmedizin BerlinBerlin, Germany; ^4^Acupuncture and Tuina School, Chengdu University of Traditional Chinese MedicineChengdu, China; ^5^Department of Radiology, Athinoula A. Martinos Center for Biomedical Imaging, Massachusetts General HospitalCharlestown, MA, USA; ^6^Department of Radiology, Logan UniversityChesterfield, MO, USA; ^7^Institute for Complementary and Integrative Medicine, University Hospital ZurichZurich, Switzerland

**Keywords:** somatosensory stimulation, functional magnetic resonance imaging (fMRI), electroencephalography (EEG), acupuncture, background rhythm, functional connectivity

## Abstract

Acupuncture can be regarded as a complex somatosensory stimulation. Here, we evaluate whether the point locations chosen for a somatosensory stimulation with acupuncture needles differently change the brain activity in healthy volunteers. We used EEG, event-related fMRI, and resting-state functional connectivity fMRI to assess neural responses to standardized needle stimulation of the acupuncture point ST36 (lower leg) and two control point locations (CP1 same dermatome, CP2 different dermatome). Cerebral responses were expected to differ for stimulation in two different dermatomes (CP2 different from ST36 and CP1), or stimulation at the acupuncture point vs. the control points. For EEG, mu rhythm power increased for ST36 compared to CP1 or CP2, but not when comparing the two control points. The fMRI analysis found more pronounced insula and S2 (secondary somatosensory cortex) activation, as well as precuneus deactivation during ST36 stimulation. The S2 seed-based functional connectivity analysis revealed increased connectivity to right precuneus for both comparisons, ST36 vs. CP1 and ST36 vs. CP2, however in different regions. Our results suggest that stimulation at acupuncture points may modulate somatosensory and saliency processing regions more readily than stimulation at non-acupuncture point locations. Also, our findings suggest potential modulation of pain perception due to acupuncture stimulation.

## Introduction

In a recent patientlevel data meta-analyses for chronic pain that included 29 randomized controlled trials, acupuncture was shown to be statistically significant superior to sham acupuncture (Vickers et al., 2012). Nevertheless, as the difference in effect size between real and sham acupuncture was small (standard mean difference of 0.15–0.23), the acupuncture point-specific effect is still controversial. One reason might be that various forms of sham acupuncture have been used in previous clinical trials. Often a penetrating sham acupuncture has been applied where either the control point location is different (Ma et al., 2012) (not a specific acupuncture point), the method of the stimulation is changed (Kleinhenz et al., 1999) (e.g., only superficial needling, no manually rotating and lift-thrusting), or both (Diener et al., 2006). Sanchez-Araujo (1998) showed in his review that studies where the control points were chosen to be near the real acupuncture points failed more frequently to show statistically significant differences between real acupuncture and sham acupuncture compared to using control acupuncture points located in different dermatomes (Sanchez-Araujo, 1998). In the past decade there has been growing interest in determining the neurophysiologic correlates of acupuncture by means of functional neuroimaging methods such as fMRI or EEG (Hui et al., 2000; Dhond et al., 2007; Hori et al., 2010; Huang et al., 2012). From a neurophysiological viewpoint, acupuncture is regarded as a complex somatosensory stimulation (Bäcker et al., 2004) and therefore is also interesting for experiments using conventional somatosensory stimulation protocols.

The purpose of this study was to evaluate whether the point locations chosen for a complex somatosensory stimulation with acupuncture needles differentially impact brain activity in healthy volunteers. We used electroencephalogram (EEG) and functional magnetic resonance imaging (fMRI) to compare standardized needle stimulation on three different point locations on the right leg: one acupuncture point (Stomach 36, ST36) and two control points that are widely accepted to not co-localize with points in any acupuncture system. One of these control points was chosen to be near the real acupuncture point in the same dermatome L5 (CP1), while the other was chosen to be in a different dermatome L2 (CP2). Imaging results are expected to differ either when comparing the two different dermatomes (CP2 different from ST36 and CP1), or when comparing the acupuncture point with the two non-acupuncture control points.

Using fMRI, the blood oxygenation level dependent (BOLD) signal can localize brain regions modulated by sensory stimulation (Bandettini et al., 1992; Frahm et al., 1992; Kwong et al., 1992; Ogawa et al., 1992; Kurth et al., 2000; Ruben et al., 2001). Several studies have demonstrated acupuncture-related brain activity changes in somatosensory and pain-related areas such as primary somatosensory cortex (S1), secondary somatosensory cortex (S2), and thalamus (Napadow et al., 2009; Huang et al., 2012). Also, functional connectivity MRI (fcMRI) revealed acupuncture-related changes in network connectivity between areas related to sensorimotor and pain processing (Dhond et al., 2008). Therefore, stimulation of the acupuncture point might specifically activate brain regions associated with pain and modulate their connectivity. Using EEG, stimulation effects can be shown by investigation of background rhythmic activity, in the somatosensory system the “mu rhythm,” respectively (Gastaut, 1952; Kuhlman, 1978). While the precise function of the mu rhythm is not clear, it is frequently referred to as an “idle rhythm” reflecting a resting-state of the somatosensory system (Ritter et al., 2009). Recent studies assume that EEG background activity is produced by inhibitory inter-neuronal activity and might reflect inhibitory top-down control (Klimesch et al., 2007; Jensen and Mazaheri, 2010). With respect to the clinical effectiveness shown for pain conditions, the stimulation of the acupuncture point might inhibit somatosensory areas and therefore increase mu-rhythmic activity.

We designed a blinded study including two series of experiments (one with EEG, one with fMRI) focusing on brain activity changes associated with complex somatosensory stimulation at one acupuncture point and two control points either close or distant to the acupuncture point. Our study was not aimed at evaluating clinical effects of acupuncture. We believe that our data provide new insights regarding cerebral processing of complex somatosensory stimulations and clarify the role of stimulus location for brain responses to complex somatosensory stimulation.

## Materials and methods

### Subjects

Twenty-three healthy subjects (11 female, 12 male, mean age 26 years, range 19–31 years) participated in the EEG experiment, and 22 healthy subjects (11 female, 11 male, mean age 26 years, range 21–32 years) participated in the fMRI experiment. Participants had no medical knowledge about acupuncture and all except one had never been treated with acupuncture before the study. Participants were informed about the needle stimulation in both experiments as follows: “… one acupuncture needle will be inserted into the muscle at three different points of the upper and lower leg….” Among all the subjects, eight participated in both EEG and fMRI experiments. All participants were right-handed (laterally score: 88.2 ± 13.4 [S.D.] over a range of −100 [fully left-handed] to 100 [fully right-handed]) according to the Edinburgh inventory (Oldfield, 1971) and gave written informed consent to participate in the experiment according to the declaration of Helsinki. The study was approved by the ethics committee of the University of Leipzig. Prior to participation all subjects underwent a comprehensive neurological examination and confirmed they were not taking any acute or chronic medication. In the EEG experiment three subjects were excluded from acupuncture because of vegetative side effects (1 sweating/male, 2 dizziness/female); in the fMRI experiment one was excluded (sweating/female).

### Detailed description of point locations

**Figure 1:**
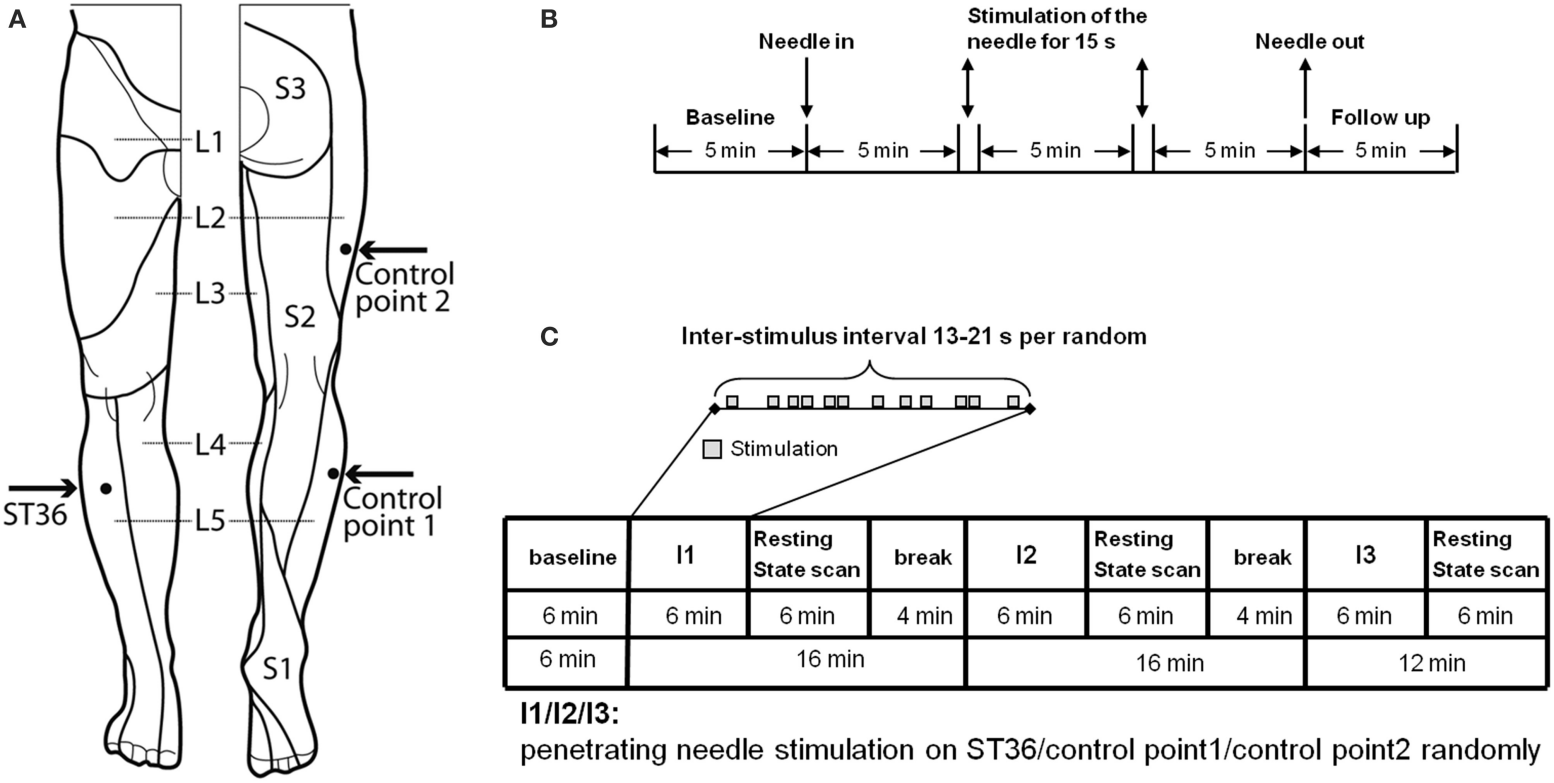
**Experimental design**. **(A)** Location of ST36 and Control points on the right leg (view from the front and from the back, figure adapted Drake et al., 2009). **(B)** Experiment design of EEG measurement. On each of the 3 randomized measurement days EEG was recorded for every subject in a sitting position, with open eyes over a 25.5-min period. After the first 5-min baseline, an acupuncture needle was inserted and was stimulated manually at a time point 5 and 10 min after insertion. After a further 5 min the needle was removed and the EEG was recorded for a further 5 min without the needle. After the measurement, individual needle sensation was measured with a questionnaire. **(C)** Experiment design of fMRI measurement. In the fMRI scanner each subject was told to lie relaxed, in a supine position, with open eyes and to concentrate on the sensation caused by the needle stimulation. After the first resting-state was scanned for 6 min, an acupuncture needle was inserted into one point according to the randomized intervention order. The needle was then immediately stimulated manually according to the event-related design. After 6 min intermittent manipulation, the needle was withdrawn and a resting-state scan was continued for 6 min, and then followed by a 4-min break for the subject without scanning. The same scan procedure was then applied for the other two interventions on the other two points. But for the third intervention, there was no 4-min break. After each post-intervention resting-state scan individual needle sensation was measured with questionnaires.

The points chosen for the intervention were developed after literature screening and a consensus process between the authors and experts of the Chengdu University of TCM, Prof Liang and Prof Li (Figure 1A).

1. Acupuncture point ST36 (Zusanli 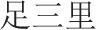):
  To locate ST36, which is placed on the anterior lower leg, the acupuncture points ST35 and ST41 are used as anatomical landmarks. ST36 is located on the line connecting ST35 with ST41, 3 B-cun inferior to ST35 (ST35 is located on the anterior aspect of the knee, in the depression lateral to the patellar ligament. ST41 is located on the anterior aspect of the ankle, in the depression at the center of the front surface of the ankle joint, between the tendons of extensor hallucis longus and extensor digitorum longus) (Who Regional Office for the Western Pacific, 2008). According to Chinese medicine theory, for healthy subjects, ST36 is a commonly used acupuncture point to strengthen Qi and blood as a health preservation application. ST36 is located on the stomach meridian within the stomach meridian area. The skin area of ST36 belongs to L5 dermatome (Yan, 2006).
2. Control point 1 (located in the same dermatome and not in the same meridian skin area):
3. The point is located lateral to the ST36 horizontally, at the middle line between Bladder meridian and Gallbladder meridian. Control point 1 is selected according to the principle of selecting non-acupuncture points from the middle line between two meridians which is commonly used in Chinese studies (Yang et al., 2009). Control point 1 is located in L5 dermatome and between Gallbladder and Bladder meridian skin area.

Control point 2 (located in another dermatome and not in the same meridian skin area):

The point is located 2 B-cun dorsally of GB31 (avoidance of bladder meridian: GB31 is located on the lateral aspect of the thigh, in the depression posterior to the iliotibial band where the tip of the middle finger rests when standing up with the arms hanging alongside the thigh). Control point 2 is already a validated non-acupuncture point used in other acupuncture studies (Brinkhaus et al., 2003; Melchart et al., 2005; Linde et al., 2006) and it is located in L2 dermatome and Gallbladder meridian skin area.

### EEG

#### Experimental procedure

All subjects received the needle stimulation of the three different points (Figure 1)—the acupuncture point ST36 in dermatome L5, the control point in the same dermatome (CP1 in L5), the control point in a different dermatome (CP2 in L2)—on 3 separate days in consecutive order. The order was randomized. Measurements were taken within 2 weeks and with at least 24 h interval between each measurement. Subjects were told to sit down in a chair in the EEG room and relax with eyes open while concentrating on the point of needling.

The penetrating needle stimulation was performed by a Chinese acupuncture physician with sterile, single use, individually wrapped acupuncture needles (0.30 × 30 mm; asia-med standard, asia-med GmbH & Co. KG, Germany). The needle was vertically inserted 1–2 cm deep into the skin depending on the size of the respective muscle on the right leg. After 5 min the needle was stimulated (manually rotating 60–90/rpm and lift-thrusting 0.3–0.5 cm for 15 s). The 15 s stimulation was repeated after 5 min without stimulation. Penetrating needle stimulation was identical for each of the three point locations (Figure 1).

#### Data acquisition

A 32-channel EEG was recorded in a noise protected and electrically shielded room using BrainAmp (Brain Product, Germany) with a sampling rate of 1000 Hz. An electrode cap (Electro-Cap International, Eaton, OH) based on the international 10–20 system was placed on the scalp. The electrode FCz was used as reference and the ground electrode was located at the sternum. Electrode impedances were less than 2 kOhm. Including the 5-min baseline, the intervention, and the follow-up measurements the EEG was recorded for 25.5 min.

After each measurement the subjects were asked to fill in the MGH Acupuncture Sensation Scale (MASS questionnaire Kong et al., 2007) to measure the subjective needle sensation.

#### Data preprocessing

For data analysis custom-built scripts in the software package Matlab (Matlab, MathWorks, Inc.) were used. Since somatosensory alpha activity (Rolandic activity) can be covered by strong occipital alpha activity, an independent component analysis (ICA) was performed to allow for a preselection of “central” ICA components. For each subject the three sessions were merged to perform one ICA calculation (FastICA algorithm in Matlab). Rolandic rhythmic activity is characterized by a central localization and a peak in the frequency spectrum in the alpha (8–15 Hz) and beta (16–30 Hz) range. Thus, ICA components were investigated for each subject and selected only if both a central topography and two peaks in the frequency spectrum were identifiable. Using this procedure, 2–10 (mean 5 ± 2 S.D.) central components were selected per subject. The selected central components were back projected and the derived dataset (now cleared from occipital alpha) was digitally filtered using a standard 3rd order band-pass Butterworth filter (low cut-off 1 Hz, high cut-off 45 Hz) and segmented into 5 epochs each lasting 4 min, based on the markers representing the interventions. Since we performed needle stimulation on the leg further data analysis was focused on electrode Cz which is located over the leg representation of S1. Frequency analysis was performed using fast Fourier transformation. The power spectral density was computed for each 4-min segment: (1) for baseline, (2) after the “needle-insertion,” (3) after the first stimulation, (4) after the second stimulation, and (5) for follow up. For the statistical analysis of the mu activity, power spectral density was averaged for the frequencies from 10 Hz to 15 Hz.

#### Statistical analysis

For Cz electrode and each condition, the mu power change and percentage change compared to baseline was analyzed using generalized linear models for our within subject design with global *F*-tests and paired *t*-tests for pair-wise comparisons between stimulation points. For our primary outcome parameter, the mean of both post-stimulation periods, a Bonferroni correction was applied to the pair-wise comparison between the three stimulation points (*p*R_corr_ = 0.05/3).

To evaluate the correlation between alpha percentage change from baseline and needle sensation (MASS Index), Spearman correlation coefficients based on the ranks of the variables were used for Cz electrode, each condition, and each needle stimulation.

The needle sensation expressed by the MASS Index was compared descriptively for the three needle stimulations at different points by presenting means and 95% confidence intervals. To test a global stimulation point effect (within-subject effect) on the MASS Index, generalized linear models (GLM) were fitted using a multivariate approach (Wilks' lambda) because sphericity was often not met. To test pair-wise differences between the three points, paired *t*-tests were used.

### fMRI

#### Experimental procedure

As shown in Figure 1C, each participant was scanned seven times (each scan 6 min): One resting-state scan in the very beginning (i.e., baseline, RS_B), then three scans with needle stimulation of one point in an event-related design, each followed by another resting-state scan (i.e., RS_ST36, RS_CP1 and RS_CP2). The three event-related scans were in randomized order over subjects. During scanning, subjects were told to remain in the supine position with eyes open while concentrating on the sensation caused by the needle stimulation. During the resting-state, participants were simply asked to keep calm and stay still with eyes open.

The penetrating needle stimulation was performed by an acupuncture physician with sterile, single use, individually wrapped needles (0.20 × 30 mm; titanium, DongBang, Acupuncture, Inc., Boryeong, Korea). The needle was first inserted 1–2 cm deep into the skin depending on the size of the muscle vertically on the right leg. The needle was manually manipulated according to the event-related design starting immediately after insertion. Auditory cues signaled the timing of the stimulation events to the acupuncturist via headphones. Each event consisted of a 3-s needle stimulation rotating 60–90/rpm and lift-thrusting 0.3–0.5 cm. The length of the inter-stimulus interval was randomized from 13 to 21 s (Figure 1B). After the event-related scan the needle was removed. Identical penetrating needle stimulation was performed on the three different point locations (Figure 1).

#### Data acquisition

fMRI Data was acquired using a 3T Siemens Verio MRI System (Siemens Medical, Erlangen, Germany) equipped for echo planer imaging with a 12-channel head coil. fMRI images were acquired using an EPI sequence (30 axial slices, in-plane resolution is 3 × 3 × 5 mm, slice thickness = 4 mm, flip angle = 90°, gap = 5 mm, repetition time = 2000 ms, echo time = 30 ms). A structural image was also acquired for each participant, using a T1-weighted MPRAGE sequence (repetition time = 12 ms, echo time = 5.65 ms, and flip angle = 19°, with elliptical sampling of k space, giving a voxel size of 1 × 1 × 1 mm). Subjects' heads were immobilized by cushioned supports, and they wore earplugs to attenuate MRI gradient noise throughout the experiment.

Within the break following resting-state scans, subjects were asked to rate the items of the MGH Acupuncture Sensation Scale (MASS questionnaire Kong et al., 2007) to measure the subjective needle sensation.

#### Data pre-processing

FMRI data pre-processing included slice time correction, head motion correction, spatial normalization to MNI152 space and spatial smoothing with a 6 mm FWHM as implemented in the SPM 8 software package (www.fil.ion.ucl.ac.uk/spm/). Individual structure T1 images were also normalized to MNI152 space and then segmented into gray matter, white matter and cerebral spinal fluid (CSF). A threshold of 0.99 was used to cut off each segmented image. For each participant, 3 mm erosion was implemented on the white matter image and 1 mm erosion on the CSF image, and these two images were then combined into one anatomical mask. We applied principal component analysis (PCA) within this CSF/white matter mask to disentangle the variance related to each fMRI dataset (3 task scans and 4 resting-state scans) using the CompCor analysis (Behzadi et al., 2007) by DPABI toolbox (toolbox for Data Processing & Analysis of Brain Imaging, http://rfmri.org/dpabi). The first five principal components together with the six head motion parameters were later applied to each individual's first level GLM as nuisance variables to regress out associated variance. A union gray matter mask (Supplemental Figure 1) was created by merging all normalized individual gray matter images. The following analyses were conducted within this average gray matter mask.

#### GLM analysis

For each subject the first-level GLM contained the three different needle stimulation conditions (i.e., ST36, CP1, CP2; 6 min stimulation for each point). For each condition, one stimulation regressor, together with the first five principal components from the CompCor analysis and six head motion parameters as nuisance regressors were included in the GLM. For the stimulation regressor, each stimulation onset was modeled with the boxcar function covering the following 3s stimulation duration. These box-car functions were convolved with the standard hemodynamic response function (HRF) as implemented in SPM 8. The long inter-stimulus intervals of 13–21 s were not explicitly modeled with the first level GLMs and hence represented an implicit baseline measure. For each of the three stimulation points we computed the individual ß-map.

On the second level (group) analyses, the main effect of each of the three stimulation points (i.e., ST36, CP1, CP2) was visualized by applying these individual ß-maps (together with age and gender as covariates) to one-sample *t*-tests to compare them against the null hypothesis. The results were corrected to the alpha-level <0.05 using AlphaSim in AFNI (Cox, 1996) (i.e., 39429 voxels within the gray matter mask, voxel-wise *p* < 0.0001, resulting cluster size >108 mm^3^). We performed a within subjects ANOVA (factorial design within SPM8) including the individual ß-maps of all three conditions (ST36, CP1, CP2) as well as age and gender as covariates to generate the inter-points comparisons (i.e., ST36—CP1, ST36—CP2 and CP1—CP2) and conjunction maps within one statistical model. Conjunction of “ST36-CP1 and ST36-CP2” was calculated to compare the activation between acupuncture and control points, conjunction of “ST36-CP2 and CP1-CP2” was calculated to compare activations of the different dermatomes (L5 vs. L2). Using AlphaSim in AFNI, the results for the interpoint-comparisons as well as for the conjunction-maps were corrected to the alpha-level < 0.05 (i.e., 39429 voxels within the gray matter mask, voxel-wise *p* < 0.01, resulting cluster size >783 mm^3^). We used “3dclust” in AFNI to detect clusters from the corrected statistical maps. All clusters that were reported in the tables are spatially separated and bigger than the volume criterion from the AlphaSim simulation analysis.

#### Functional connectivity analyses

For each of the four resting-state scans (RS_B, RS_ST36, RS_CP1, and RS_CP2) the first 10 volumes were discarded to account for the saturation of the BOLD response. Temporal band-pass filtering (0.01–0.08 Hz) and removal of linear trend was performed by the REST toolbox (www.restfmri.net). Seed-based voxel-wise functional connectivity analysis was performed for each resting-state scan using region of interest (ROI) spheres with 6 mm radius as seeds. As a proof of concept, one seed was placed on posterior cingulate cortex (PCC, Talairach space, *x* = −2, *y* = −36, *z* = 37 from a previous study Fox et al., 2005) that, as hypothesized, revealed the default mode network (Supplemental Figure 1). The other seed was derived from a group-level one-sample *t*-test across all three stimulation points together, with the maximum found at the parietal operculum as the anatomical site of S2 (Talairach space, *x* = −54, *y* = −21, *z* = 21).

Then, the average time course within the ROI was extracted as the seed signal, and a voxel-wise temporal correlation analysis was performed across all voxels within the averaged gray matter mask for each individual resting-state scan. The correlation maps were transferred to Fisher's z maps for further statistical analysis (Greicius et al., 2007). First, a one-sample *t*-test against null hypothesis was performed on the spatial correlation maps of each resting-state scan (together with age and gender as covariates) for the PCC-seed and the S2-seed, respectively. The results were corrected to the alpha-level <0.05 using AlphaSim in AFNI (i.e., 39429 voxels within the gray matter mask, voxel-wise *p* < 0.0001, resulting cluster size >108 mm^3^). Again, we performed a within subjects ANOVA including the individual spatial correlation maps of all four resting-state scans as well as age and gender as covariates. Within this model, we generated the comparisons to baseline (RS_ST36-RS_B, RS_CP1-RS_B, and RS_CP2-RS_B), the inter-points comparisons (RS_ST36-RS_CP1, RS_ST36-RS_CP2, and RS_CP1-RS_CP2), and the conjunction maps. Conjunction of “RS_ST36-RS_CP1 and RS_ST36-RS_CP2” was calculated to compare functional connectivity between acupuncture and control points, conjunction of “RS_ST36-RS_CP2 and RS_CP1-RS_CP2” was calculated to compare connectivity between the different dermatomes (L5 vs. L2). Using AlphaSim in AFNI, the results for the different comparisons as well as for the conjunction-maps were corrected to the alpha-level <0.05 (i.e., 39429 voxels within the gray matter mask, voxel-wise *p* < 0.01, resulting cluster size >783 mm^3^). As described above, we used “3dclust” in AFNI to detect clusters from the corrected statistical maps.

#### Needle sensation analyses

The needle sensation expressed by the MASS Index was compared descriptively for the three needle stimulations at different points by presenting means and 95% confidence intervals. To test a global stimulation point effect (within-subject effect) on MASS Index, generalized linear models (GLM) were fitted using a multivariate approach (Wilks' lambda) because sphericity was often not met. To test pair-wise differences between the three points, paired *t*-tests were used.

For each region that was detected in the conjunction analyses (Figures 3C,D), Pearson correlation coefficients were calculated across participants between the mean beta value across voxels within the respective region and the MASS index of each stimulation point.

## Results

### EEG

#### Mu rhythm

**Figure 2:**
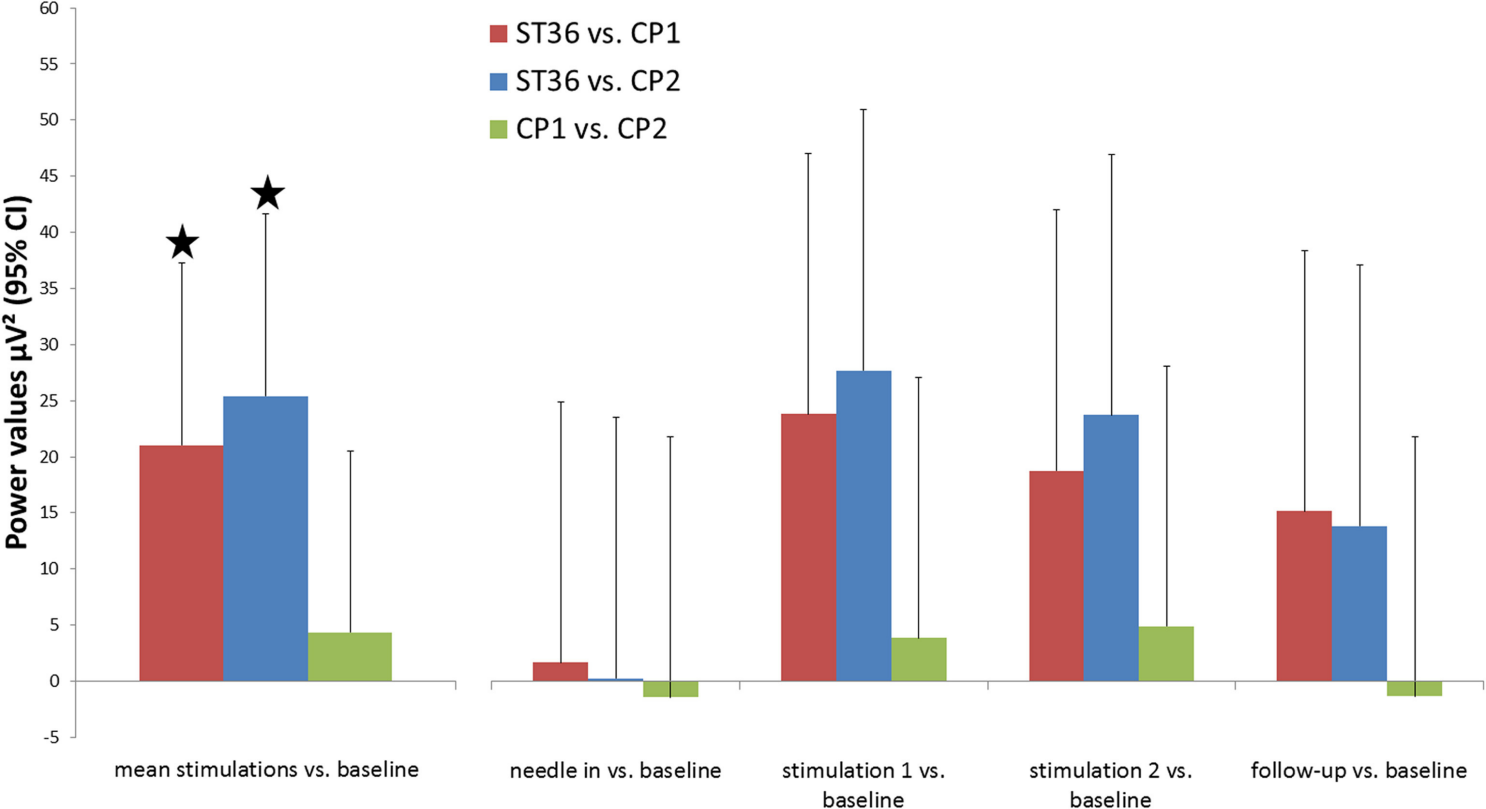
**Changes in background rhythm strength in the comparison of the three point locations**. Power spectral density was calculated for segments of 4 min in the middle of each measurement block (baseline, needle-in, stimulation 1, stimulation 2, follow-up) and power values for frequencies 10–15 Hz (μ-rhythm) were averaged. The primary outcome parameter, the mean of both post-stimulation blocks, is highlighted on the left. Results are shown for electrode Cz (located over the leg representation of the somatosensory cortex). ^*^ Indicate significant differences after Bonferroni correction.

The mu rhythm is one of the important human brain background rhythms and is associated with the primary somatosensory area, thus having a central topography (Salmelin and Hari, 1994). In healthy volunteers we stimulated the three different points mentioned above in the same manner and compared their respective influences on mu rhythm. Data is shown for electrode Cz which is closest to the lower limb representation in S1. Mu rhythm power was significantly enhanced after stimulation of ST36 compared to the stimulation of the two control points (mean of stimulation phase 1 and phase 2 vs. baseline: ST36 vs. CP1 21.02 μV^2^ 95%CI [4.78;37.27], *p* = 0.012, ST36 vs. CP2 25.38 [9.12;41.65], *p* = 0.003, significance level Bonferroni corrected 0.05/3). Comparison of mu rhythm for the two control points found no significant differences (CP2 vs. CP1 −4.36 [−20.53;11.81], *p* = 0.598, Figure 2).

#### Needle sensation

As our results for the mu rhythm may have been influenced by differences in needle sensation, the evoked sensation was measured using the MGH Acupuncture Sensation Scale (MASS Kong et al., 2007).

The MASS Index was used as a measure of needle sensation for ST36, CP1, and CP2 (3.15 [2.00;4.30], 3.37 [2.47;4.28], and 1.81 [1.13;2.50], respectively). Comparisons of ST36 vs. CP2 and CP1 vs. CP2 were statistically different (pairwise *t*-test, *p* = 0.034 and *p* < 0.001, respectively). However, a significant difference was not found between ST36 and CP1 (*p* = 0.674).

No correlations were found when exploring the relationship between the MASS index and the percentage change of mu rhythm power (all *r*-values between −0.16 and 0.40 with *p* > 0.080).

### fMRI

#### Stimulation scans

**Figure 3:**
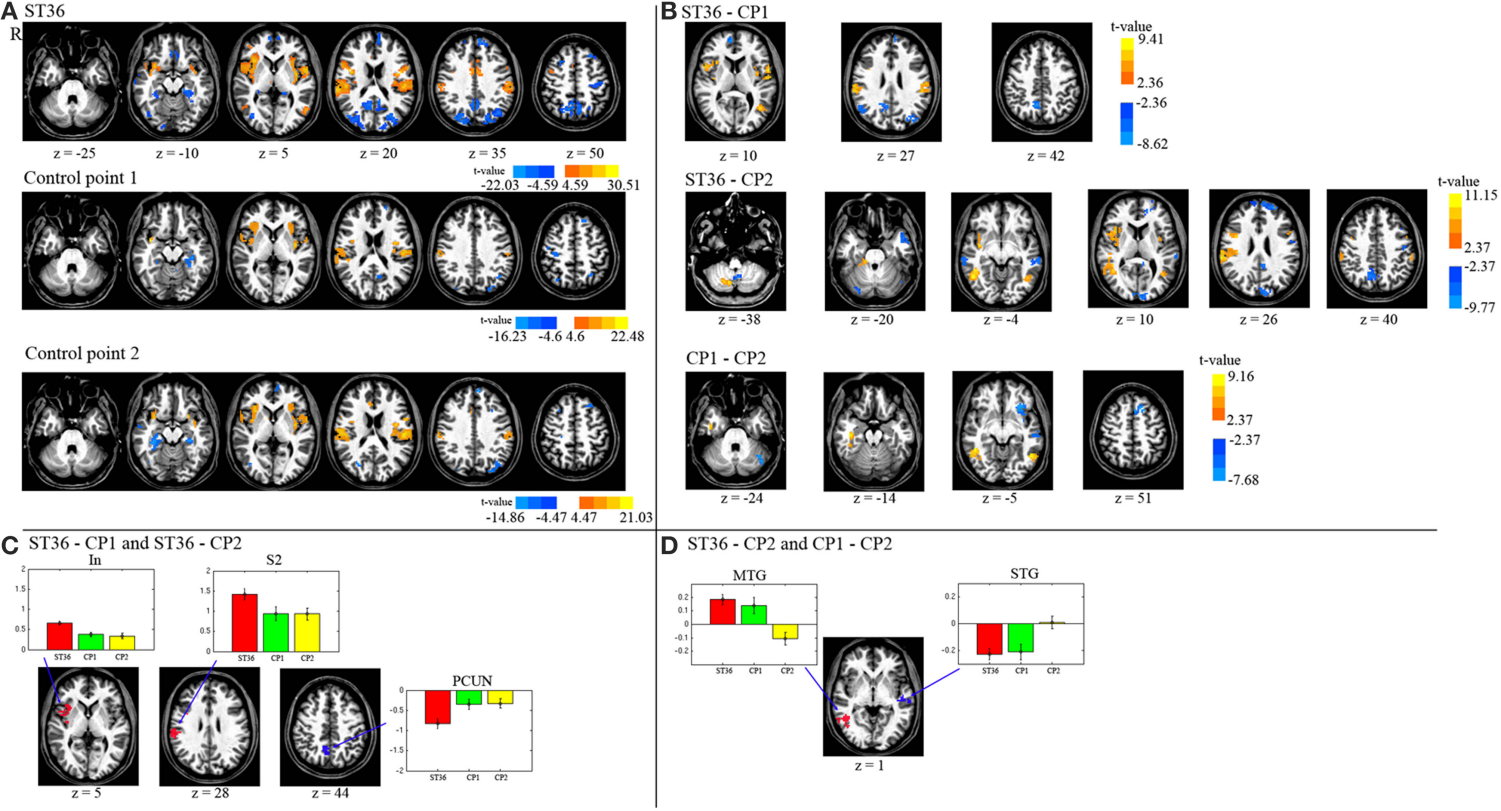
**(A)** Displayed are the activation and deactivation for the different point stimulations (group-level t-maps, *P* < 0.05, corrected). **(B)** The de/activation contrasts between the three different point locations are presented (*P* < 0.05, corrected). **(C)** The conjunction map of the 1st and 2nd row of part **(B)** (acupuncture vs. control points). **(D)** The conjunction map of the 2nd and 3rd row of part **(B)** (dermatome L5 vs. L2). The barplots show the beta values across participants (average and standard error) within the respective region. R means right hemisphere. Talairach z coordinates are displayed. positive values: red, negative: blue.

The results of the intra-point analysis summarizing the BOLD response to needle stimulation of ST36 and the two control points are shown in Figure 3A and Table 1. For all three point stimulations we found significant activation in bilateral insula/S2 and left inferior semi-lunar lobule and deactivation in bilateral precuneus, right middle temporal gyrus, left superior frontal gyrus, right precentral gyrus, left medial frontal gyrus, right paracentral lobule, and bilateral parahippocampal gyrus.

**Table 1:** **Foci with significant BOLD response from the three points (*P* < 0.05, corrected)**.

**Task**	**Area**	**Left/Right hemisphere**	**Brodmann areas**	**Talairach space, x,y,z**	***T*–value**	***p*–value**	**Volume (mm^3^)**
**ST36**	Insula/SII	L	13	−36, −3, 17	30.51	1.72E-17	28431
	Insula	R	13	36, −5, −2	28.78	5.12E-17	21708
	Precuneus	L	31	−20, −73, 25	−21.57	1.05E-14	12555
	Middle temporal gyrus	R	19	30, −76, 22	−20.61	2.41E-14	11016
	SII	R	40	55, −24, 22	26.74	2.00E-16	10908
	Precuneus	R	7	11, −56, 46	−17.50	4.63E-13	9045
	Precuneus	R	7	−3, −62, 52	−20.18	3.54E-14	7668
	Superior frontal gyrus	L	9	−8, 56, 25	−15.54	3.87E-12	5211
	Parahippocampal gyrus	L	37	−28, −47, −7	−13.85	2.89E-11	2835
	SMA	L	24	−8, −3, 38	17.73	3.67E-13	2835
	SMA	R	24	6, 6, 30	18.36	1.96E-13	2673
	Precentral gyrus	L	4	−36, −16, 49	−12.48	1.74E-10	1971
	Inferior temporal gyrus	R	19	39, −70, 1	−12.92	9.60E-11	1917
	Parahippocampal gyrus	R	36	30, −36, −7	−13.34	5.59E-11	1485
	Medial frontal gyrus	L	11	−6, 25, −15	−12.88	1.02E-10	1242
	Inferior semi-lunar lobule	L	/	−17, −67, −36	14.25	1.78E-11	1215
	Middle temporal gyrus	L	37	−53, −62, 6	14.47	1.36E-11	1188
	Inferior semi-lunar lobule	R	/	11, −67, −41	16.03	2.22E-12	1134
	Middle frontal gyrus	R	9	25, 22, 41	−12.63	1.42E-10	1107
	Paracentral Lobule	R	6	6, −30, 68	−18.25	2.19E-13	1026
	Superior temporal gyrus	R	21	61, −21, 1	−14.97	7.46E-12	864
	Precentral gyrus	R	4	25, −21, 60	−15.75	3.03E-12	837
	Medial frontal gyrus	R	25	11, 31, −15	−12.41	1.92E-10	567
	Medial frontal gyrus	R	10	6, 53, 6	−12.86	1.05E-10	513
	Middle temporal gyrus	L	21	−53, 3, −18	−13.52	4.39E-11	486
	Lingual gyrus	L	19	−22, −67, 1	−11.45	7.47E-10	486
	Thalamus	L	/	−11, −36, 9	−12.22	2.48E-10	486
	Supramarginal gyrus	R	39	50, −53, 25	−11.33	8.85E-10	432
	Middle frontal gyrus	R	6	39, 0, 46	11.88	4.00E-10	432
	Superior frontal gyrus	L	8	−25, 28, 49	−12.64	1.39E-10	405
	Precentral gyrus	R	6	17, −19, 68	−13.95	2.58E-11	405
	Postcentral gyrus	L	5	−17, −44, 68	12.27	2.31E-10	378
	Inferior frontal gyrus	R	46	41, 42, 4	12.34	2.10E-10	351
	Cerebellar Tonsil	L	/	−31, −59, −41	13.77	3.21E-11	324
	Cerebellar Tonsil	R	/	3, −53, −38	−11.22	1.05E-09	297
	Pyramis	R	/	44, −64, −31	−10.85	1.81E-09	297
	Middle temporal gyrus	R	37	44, −56, 4	10.72	2.21E-09	297
	Precuneus	R	7	25, −53, 54	−14.91	7.95E-12	270
	Middle temporal gyrus	R	21	58, −5, −12	−11.66	5.48E-10	243
	Superior temporal gyrus	L	21	−59, −24, 1	−11.00	1.46E-09	243
	Lingual gyrus	R	18	14, −88, −10	−10.40	3.62E-09	216
	Middle temporal gyrus	R	21	61, −11, −12	−10.39	3.72E-09	189
	Thalamus	R	/	6, −16, 9	11.49	6.99E-10	189
	Superior temporal gyrus	R	38	44, 8, −10	11.23	1.03E-09	162
	Middle occipital gyrus	L	19	−34, −70, 1	−10.70	2.30E-09	162
	Superior frontal gyrus	R	8	17, 48, 38	−12.31	2.19E-10	162
	Superior frontal gyrus	R	6	8, 3, 65	10.72	2.22E-09	162
	Caudate	R	/	0, 14, 1	−12.13	2.81E-10	108
	Postcentral gyrus	R	40	61, −19, 20	14.42	1.43E-11	108
	Anterior cingulate	R	24	6, 25, 22	10.15	5.43E-09	108
	Precuneus	L	7	−17, −70, 46	−10.86	1.79E-09	108
	Precuneus	L	7	−22, −56, 49	−10.01	6.72E-09	108
CP1	Insula	R	38	36, 0, −7	22.48	4.91E-15	8613
	Insula	L	38	−34, −8, 4	16.14	1.96E-12	7020
	SII	R	2	55, −19, 25	17.84	3.28E-13	6264
	SII	L	13	−48, −19, 20	15.99	2.33E-12	4428
	Parahippocampal gyrus	L	30	−25, −38, −2	−15.45	4.28E-12	2160
	Postcentral gyrus	R	30	33, −21, 46	−13.00	8.65E-11	1323
	Inferior semi-lunar lobule	L	/	−11, −67, −41	14.62	1.12E-11	1134
	Precuneus	L	7	−22, −76, 38	−11.76	4.74E-10	729
	Precentral gyrus	R	6	19, −16, 68	−12.98	8.93E-11	540
	Parahippocampal gyrus	L	35	−28, −19, −15	−10.91	1.67E-09	432
	Middle temporal gyrus	L	21	−56, −21, −4	−11.23	1.03E-09	432
	Angular gyrus	L	40	−39, −56, 36	−11.06	1.33E-09	405
	Paracentral Lobule	R	6	6, −33, 62	−11.85	4.21E-10	405
	Parahippocampal gyrus	R	19	33, −38, −2	−16.07	2.13E-12	378
	Superior frontal gyrus	L	10	−20, 53, 20	−10.36	3.86E-09	324
	Posterior cingulate	L	31	−8, −56, 20	−10.44	3.43E-09	270
	Superior frontal gyrus	L	8	−14, 31, 49	−10.93	1.61E-09	243
	Medial frontal gyrus	L	8	−6, 48, 41	−10.97	1.52E-09	216
	Precuneus	L	7	−25, −53, 49	−10.66	2.44E-09	216
	Superior Parietal Lobule	R	7	28, −62, 52	−11.23	1.02E-09	189
	Posterior Cingulate	R	30	8, −53, 14	−9.83	9.08E-09	162
	Cingulate gyrus	R	24	3, 3, 30	10.79	2.00E-09	162
	Parahippocampal gyrus	R	36	25, −36, −12	−10.19	5.05E-09	135
	Middle occipital gyrus	R	19	36, −70, 9	−9.99	6.93E-09	135
	Precuneus	R	19	30, −73, 33	−10.66	2.44E-09	135
	Culmen	L	/	−6, −47, −10	−10.97	1.52E-09	108
	Middle temporal gyrus	R	7	30, −64, 28	−10.02	6.69E-09	108
	Precentral gyrus	R	4	44, −11, 49	−11.41	7.95E-10	108
CP2	SII/Insula	L	13	−50, −19, 22	20.91	1.84E-14	22491
	Insula/SII	R	13	33, 20, −2	21.03	1.66E-14	8559
	Middle occipital gyrus	R	37	36, −67, 1	−14.44	1.40E-11	8019
	Postcentral gyrus	R	3	58, −21, 38	17.67	3.89E-13	7182
	Precuneus	L	19	−28, −79, 36	−11.90	3.92E-10	1998
	Inferior semi-lunar lobule	L	/	−14, −67, −38	16.18	1.88E-12	972
	Superior frontal gyrus	L	9	−8, 50, 33	−12.76	1.19E-10	810
	Middle temporal gyrus	R	39	41, −56, 25	−12.81	1.12E-10	756
	Superior frontal gyrus	L	8	−25, 28, 49	−11.26	9.88E-10	729
	Parahippocampal gyrus	L	27	−25, −30, −7	−12.50	1.69E-10	594
	Cingulate gyrus	L	23	−6, −16, 30	12.30	2.23E-10	567
	Medial frontal gyrus	R	6	6, 14, 44	11.15	1.15E-09	567
	Middle temporal gyrus	R	21	52, −8, -12	−11.80	4.49E-10	513
	Medial frontal gyrus	L	10	−6, 56, 6	−11.43	7.64E-10	405
	Anterior cingulate	R	24	6, 25, 22	11.90	3.92E-10	378
	Cuneus	R	19	30, −73, 28	−10.74	2.15E-09	378
	Paracentral lobule	R	6	6, −33, 60	−13.62	3.86E-11	324
	Precuneus	R	31	25, −67, 20	−12.91	9.72E-11	243
	Middle frontal gyrus	R	8	25, 17, 46	−11.13	1.19E-09	243
	Cingulate gyrus	R	23	6, −16, 30	11.10	1.25E-09	216
	Cingulate gyrus	R	24	6, 11, 28	9.89	8.15E-09	216
	Cingulate gyrus	L	24	−3, 0, 38	10.35	3.94E-09	216
	Parahippocampal gyrus	R	34	28, −11, −15	−11.96	3.60E-10	189
	Middle frontal gyrus	L	8	−31, 20, 38	−11.38	8.30E-10	189
	Postcentral gyrus	R	3	22, −24, 49	−10.99	1.47E-09	162
	Declive	R	/	17, −67, −15	10.20	4.97E-09	135
	Precentral gyrus	R	4	14, −24, 68	−11.34	8.71E-10	135
	Putamen	L	/	−25, 8, −7	16.29	1.68E-12	108
	Caudate	R	/	8, 6, 12	10.29	4.32E-09	108

*Displayed are voxels of maximal significance. If the activated area crosses the midline, only the side of the highest value is displayed. The coordinates are in the Talairach space. T and p value is on the voxel-level*.

We compared BOLD responses of the different points (ST36 vs. CP1, ST36 vs. CP2, and CP1 vs. CP2, shown in Figure 3B and Table 2) and with a conjunction analysis we evaluated shared areas for the comparison of acupuncture point vs. control points (conjunction of ST36-CP1 and ST36-CP2) and the comparison of two different dermatomes L5 and L2 (conjunction of ST36-CP2 and CP1-CP2).

**Table 2:** **BOLD changes: a comparison of the three points showing all significant contrasts (*P* < 0.05, corrected)**.

	**Area**	**Left/Right hemisphere**	**Brodmann areas**	**Talairach space, x,y,z**	***T*-value**	***p*–value**	**Volume (mm^3^)**
ST36-CP1	Insula	R	44	47, 8, 14	9.42	1.79E-08	7020
	SII	L	43	−53, −16, 20	8.68	6.33E-08	6777
	SII	R	40	58, −21, 25	7.64	4.26E-07	3294
	Angular gyrus	L	19	−36, −73, 33	−7.59	4.76E-07	2322
	Insula	L	13	−34, 0, −2	8.70	6.12E-08	2214
	Medial frontal gyrus	R	10	6, 50, 9	−7.67	4.02E-07	1755
	Medial frontal gyrus	L	9	−6, 50, 20	−7.10	1.23E-06	1701
	Precuneus	R	7	8, −53, 44	−8.26	1.34E-07	1404
	Angular gyrus	R	39	47, −64, 33	−6.42	4.77E-06	1350
	Insula	L	13	−34, −3, 12	7.12	1.18E-06	1323
	Posterior cingulate	R	23	11, −50, 25	−6.44	4.57E-06	1269
	Middle temporal gyrus	L	19	−39, −59, 12	7.06	1.32E-06	1026
ST36-CP2	Insula	R	13	36, 0, 1	11.15	1.16E-09	11232
	Middle temporal gyrus	R	37	47, −53, −4	9.78	9.84E-09	7641
	Cuneus	L	18	−3, −93, 12	−9.68	1.16E-08	5616
	SII	R	40	61, −30, 25	9.27	2.31E-08	5022
	Superior frontal gyrus	L	10	−22, 50, 25	−7.96	2.35E-07	4536
	Superior temporal gyrus	L	22	−48, −13, 1	−9.01	3.60E-08	3699
	Precuneus	R	7	8, −56, 44	−8.23	1.42E-07	2646
	Middle temporal gyrus	L	37	−42, −56, 1	9.08	3.16E-08	2565
	Middle temporal gyrus	L	21	−39, 3, −28	−7.42	6.49E-07	2025
	Inferior Parietal Lobule	L	40	−56, −30, 38	7.72	3.70E-07	1998
	Cerebellar Tonsil	L	/	−42, −64, −28	−6.85	2.00E-06	1944
	Inferior semi-lunar lobule	R	/	11, −64, −38	8.77	5.46E-08	1431
	Precentral gyrus	L	6	−48, −11, 30	−6.62	3.20E-06	1404
	Middle temporal gyrus	R	21	61, −27, -10	−6.95	1.63E-06	1377
	Precuneus	L	7	−3, −59, 46	−6.47	4.36E-06	1296
	Inferior frontal gyrus	L	9	−50, 8, 33	9.70	1.11E-08	1269
	Superior frontal gyrus	R	10	11, 62, 22	−6.85	2.02E-06	1242
	Culmen	R	/	17, −30, −18	8.92	4.20E-08	1188
	Posterior cingulate	L	29	−8, −41, 12	−8.67	6.54E-08	1134
	Medial frontal gyrus	L	32	−11, 39, 14	−7.25	9.17E-07	972
	Cerebellar Tonsil	L	/	−6, −50, −44	−6.59	3.41E-06	837
	Declive	R	/	28, −76, −20	−5.81	1.71E-05	837
	Insula	L	13	−39, 3, 1	6.89	1.85E-06	837
	Precentral gyrus	L	9	−39, 20, 36	−6.48	4.28E-06	810
CP1-CP2	Middle temporal gyrus	R	37	44, −47, −2	8.74	5.70E-08	3861
	Superior temporal gyrus	L	22	−48, −21, 1	−6.88	1.90E-06	1917
	Inferior frontal gyrus	L	47	−31, 20, −2	−7.41	6.60E-07	1890
	Superior frontal gyrus	L	6	−14, 17, 52	−7.29	8.41E-07	1539
	Middle temporal gyrus	L	37	−48, −53, −2	9.16	2.76E-08	1485
	Parahippocampal gyrus	R	36	39, −21, −15	8.37	1.11E-07	1323
	Tuber	L	/	−42, −67, −25	−7.66	4.12E-07	1134
	Parahippocampal gyrus	R	36	36, −33, −10	6.79	2.27E-06	918
ST36-CP1 and ST36-CP2	Insula	R	13	/	positive	/	5346
	SII	R	42	/	positive	/	2214
	Precuneus / PCC	R	7	/	negative	/	1134
ST36-CP2 and CP1-CP2	Middle temporal gyrus	R	37	/	positive	/	2916
	Superior temporal gyrus	L	21	/	negative	/	810

*Displayed are voxels of maximal significance. If the activated area crosses the midline, only the side of the highest value is displayed. The coordinates are in the Talairach space. T and p value is on the voxel-level. The results of the conjunction analysis are also listed. “Positive” means ST36 presented higher activity than two control points, and vice versa*.

For the comparison between acupuncture point and control points (ST36-CP1 and ST36-CP2), the conjunction analysis (Figure 3C, Table 2) revealed that right insula and right S2 presented higher activation during stimulation of ST36. The right precuneus/posterior cingulate cortex (PCC) presented pronounced deactivation during stimulation of the acupuncture point.

For the comparison between the dermatomes (ST36-CP2 and CP1-CP2), a common positive contrast was shown for right middle temporal gyrus (MTG) due to deactivation during stimulation of CP2 compared to activation when stimulating the other two points (Figure 3D, Table 2). Left superior temporal gyrus (STG) presented pronounced deactivation when stimulating ST36 or CP1 compared to stimulation of CP2.

#### Resting-state scans

At the first stage, the default mode network and somatosensory network were detected via the seed-based correlation analysis within the resting-state scans. By visual inspection, PCC, mPFC and bilateral angular gyrus (prominent marker of the default mode network) were all found in the PCC-seed-based correlation analysis (Supplemental Figure 1). For the S2-seed-based correlation analysis, we found bilateral S2, supplementary motor area (SMA), and bilateral S1/M1 as prominent areas corresponding to the somatosensory network (Supplemental Figure 1, Table 3). The results of the S2-seed-based correlation analysis were further compared between the different resting-state scans.

**Table 3:** **Brain regions which were detected on the group level in the somatosensory network analysis from all resting-state sessions (*P* < 0.05, corrected)**.

**Resting-state**	**Area**	**Left/Right hemisphere**	**Brodmann areas**	**Talairach space, x,y,z**	***T*-value**	***p*-value**	**Volume (mm^3^)**
RS_ST36	SII	L	40	−50, −19, 22	66.37	7.68E-24	66285
	SII	R	40	58, −19, 25	28.07	8.12E-17	43983
	Inferior semi-lunar lobule	L	/	−17, −62, −41	24.14	1.33E-15	10854
	Medial frontal gyrus/SMA	R	6	11, −5, 62	17.59	4.23E-13	7425
	Culmen	R	/	14, −47, −12	16.94	8.31E-13	5211
	Middle temporal gyrus	L	37	−48, −64, 9	15.56	3.75E-12	4158
	Middle temporal gyrus	R	37	52, −59, 6	17.26	5.93E-13	2538
	Precuneus	L	39	−39, −67, 36	−14.47	1.35E-11	1998
	Precuneus	L	31	−14, −44, 33	−12.90	9.94E-11	1107
	Cingulate gyrus	R	31	11, −21, 41	12.54	1.61E-10	1107
	Precuneus	R	7	14, −47, 57	13.11	7.47E-11	1080
	Inferior Parietal Lobule	R	40	44, −59, 38	−14.94	7.69E-12	1026
	Culmen	L	/	−17, −50, −18	11.93	3.72E-10	648
	Superior frontal gyrus	L	9	−14, 45, 36	−11.76	4.79E-10	432
	Fusiform gyrus	L	37	−42, −44, −12	11.39	8.19E-10	297
	Parahippocampal gyrus	L	19	−34, −59, −4	10.10	5.87E-09	243
	Middle frontal gyrus	L	6	−20, 22, 54	−11.11	1.23E-09	243
	Middle frontal gyrus	R	6	19, −16, 60	14.61	1.14E-11	243
	Anterior cingulate	R	32	6, 36, 25	−10.89	1.71E-09	216
	Fusiform gyrus	L	20	−39, −21, −23	11.49	6.99E-10	162
	Middle temporal gyrus	R	39	41, −62, 17	9.90	8.10E-09	162
	Postcentral gyrus	R	2	28, −36, 60	10.55	2.86E-09	162
	Declive	L	/	−22, −59, −15	9.99	6.97E-09	135
	Precentral gyrus	L	6	−14, −21, 68	13.06	8.02E-11	135
	Fusiform gyrus	R	37	41, −41, −12	10.22	4.82E-09	108
	Postcentral gyrus	R	3	44, −24, 54	11.69	5.24E-10	108
	Postcentral gyrus	L	3	−42, −27, 54	11.42	7.74E-10	108
RS_CP1	SII	L	43	−50, −19, 20	83.81	9.26E-26	71010
	SII	R	43	50, −16, 14	28.31	6.94E-17	47142
	Medial frontal gyrus / SMA	R	32	6, 6, 44	29.27	3.74E-17	9153
	Inferior semi-lunar lobule	L	/	−14, −62, −44	18.80	1.28E-13	3672
	Inferior semi-lunar lobule	R	/	11, −64, −44	14.60	1.15E-11	2970
	Precuneus	L	19	−31, −73, 38	−16.54	1.28E-12	2484
	Inferior Parietal Lobule	R	40	41, −59, 41	−11.78	4.66E-10	2376
	Culmen	R	/	19, −53, −18	14.49	1.32E-11	1944
	Middle temporal gyrus	L	39	−45, −53, 6	13.05	8.06E-11	1944
	Culmen	L	/	−17, −56, −18	14.46	1.37E-11	1836
	Middle temporal gyrus	R	19	50, −53, 4	12.99	8.80E-11	1782
	Postcentral gyrus	R	3	19, −36, 60	18.09	2.57E-13	1674
	Cingulate gyrus	R	31	14, −24, 41	12.75	1.21E-10	999
	Middle frontal gyrus	R	8	39, 25, 38	−13.82	3.02E-11	972
	Culmen	L	/	−22, −44, −23	12.08	3.04E-10	324
	Parahippocampal gyrus	R	27	30, −30, −7	−13.15	7.13E-11	270
	Middle frontal gyrus	R	46	47, 31, 17	−10.71	2.25E-09	216
	Middle frontal gyrus	L	6	−31, 17, 54	−14.02	2.34E-11	189
	Precuneus	R	19	33, −73, 33	−10.46	3.32E-09	162
	Postcentral gyrus	L	3	−42, −27, 54	15.10	6.37E-12	162
	Inferior frontal gyrus	R	47	28, 28, −2	11.21	1.06E-09	135
	Middle frontal gyrus	R	11	25, 48, −10	13.20	6.65E-11	108
	Middle temporal gyrus	L	39	−56, −56, 9	10.50	3.14E-09	108
	Precuneus	L	31	−6, −59, 30	−9.79	9.73E-09	108
	Superior frontal gyrus	R	8	17, 22, 46	−9.76	1.01E-08	108
	Postcentral gyrus	R	3	28, −33, 57	11.33	8.85E-10	108
RS_CP2	SII	L	43	−50, −19, 20	79.22	2.69E-25	56430
	SII	R	43	58, −19, 22	35.20	1.19E-18	47061
	SMA	L	24	−3, 3, 41	24.16	1.31E-15	11718
	SMA	R	24	6, 6, 44	22.23	6.05E-15	5940
	Inferior semi-lunar lobule	R	/	14, −64, −44	17.17	6.54E-13	3078
	Middle temporal gyrus	L	39	−45, −53, 9	14.47	1.35E-11	2079
	Fusiform gyrus	L	37	−39, −47, −15	22.08	6.84E-15	1971
	Fusiform gyrus	R	20	41, −38, −12	16.87	8.99E-13	1593
	Cerebellar Tonsil	L	/	−25, −53, −46	14.16	1.97E-11	1431
	Parahippocampal gyrus	R	34	19, 0, −12	20.29	3.20E-14	756
	Postcentral gyrus	R	40	22, −38, 57	11.41	7.88E-10	756
	Cingulate gyrus	R	31	11, −21, 41	14.04	2.29E-11	540
	Inferior semi-lunar lobule	L	/	−11, −64, −44	15.10	6.37E-12	432
	Paracentral Lobule	L	31	−8, −27, 44	14.41	1.46E-11	405
	Middle occipital gyrus	R	37	47, −64, −7	11.02	1.40E-09	324
	Middle temporal gyrus	L	20	−31, 0, −33	10.74	2.14E-09	243
	Parahippocampal gyrus	L	36	−39, −24, −15	12.39	1.98E-10	189
	Culmen	R	/	33, −38, −25	10.67	2.40E-09	162
	Middle frontal gyrus	L	10	−31, 39, 14	10.72	2.21E-09	135
	Caudate	R	/	14, 11, 17	−11.89	3.97E-10	135
	Superior frontal gyrus	L	6	−17, −3, 68	10.80	1.98E-09	135
	Fusiform gyrus	L	37	−36, −59, −12	10.10	5.83E-09	108
RS_B	SII	L	40	−50, −19, 22	52.90	5.59E-22	61398
	SII	R	40	44, −19, 22	22.59	4.49E-15	59967
	Cerebellar Tonsil	R	/	14, −59, −41	19.38	7.38E-14	4752
	Inferior semi-lunar lobule	L	/	−17, −62, −38	13.82	3.01E-11	2673
	Culmen	L	/	−28, −47, −23	13.74	3.34E-11	2079
	Declive	R	/	19, −62, −12	13.86	2.87E-11	1485
	Middle temporal gyrus	L	39	−50, −67, 9	10.89	1.72E-09	1323
	Middle temporal gyrus	R	37	52, −59, 1	12.30	2.22E-10	1242
	Paracentral Lobule	L	5	−6, −33, 57	14.58	1.18E-11	702
	Angular gyrus	L	39	−39, −62, 33	−10.96	1.55E-09	594
	Cerebellar Tonsil	L	/	−28, −44, −41	11.29	9.41E-10	432
	Cingulate gyrus	R	31	17, −41, 33	−11.05	1.34E-09	432
	Middle frontal gyrus	R	8	36, 17, 44	−10.61	2.62E-09	324
	Inferior frontal gyrus	L	10	−31, 36, 14	10.16	5.32E-09	297
	Precuneus	L	31	−11, −53, 30	−10.27	4.50E-09	297
	Precentral gyrus	R	4	33, −16, 52	11.75	4.86E-10	297
	Subcallosal gyrus	R	34	14, 6, −12	11.44	7.60E-10	270
	Putamen	R	/	25, 11, 6	11.95	3.63E-10	216
	Postcentral gyrus	L	4	−17, −33, 62	11.91	3.83E-10	216
	Postcentral gyrus	L	2	−22, −36, 65	15.53	3.91E-12	216
	Cingulate gyrus	L	31	−3, −30, 36	−11.65	5.54E-10	189
	Culmen	R	/	14, −44, −15	11.08	1.28E-09	162
	Putamen	L	/	−22, 11, 4	10.41	3.58E-09	162
	Supramarginal gyrus	R	39	39, −53, 28	−9.84	8.93E-09	162
	Cingulate gyrus	R	31	3, −33, 36	−10.63	2.55E-09	135
	Postcentral gyrus	L	4	−36, −27, 57	10.11	5.75E-09	135
	Middle temporal gyrus	L	22	−36, −53, 9	10.59	2.69E-09	108
	Claustrum	L	13	−25, 22, 9	11.09	1.27E-09	108
	Superior frontal gyrus	R	9	30, 34, 30	9.81	9.36E-09	108
	Postcentral gyrus	R	4	14, −41, 65	11.04	1.37E-09	108
	Precentral gyrus	L	4	−22, −24, 65	10.82	1.90E-09	108

*Displayed are voxels of maximal significance. If the activated area crosses the midline, only the side of the highest value is displayed. The coordinates are in the Talairach space. T and p value is on the voxel-level*.

**Figure 4:**
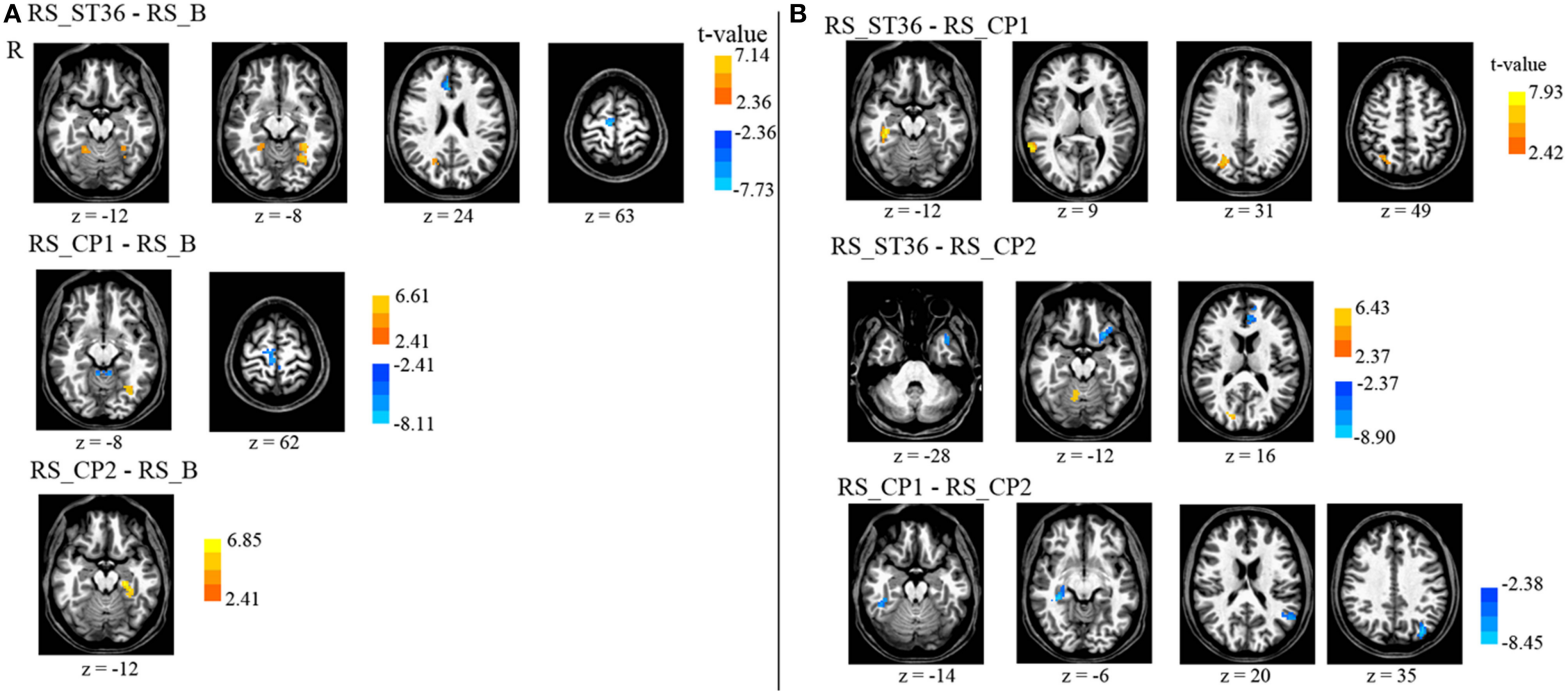
**Comparison of the seed-based somatosensory resting-state connectivity between all resting-state sessions (*P* < 0.05, corrected)**. **(A)** The comparison of post-stimulation resting-state scans with the baseline scan. **(B)** The comparison of the post-stimulation resting-state scans with each other. There is no cluster surviving the conjunction analyses across the first and second row (acupuncture vs. control points) as well as across the second and the third row (dermatome L5 vs. L2). R means right hemisphere. Talairach z coordinates are displayed. positive values: red, negative: blue.

The different brain areas that showed changes in functional connectivity after stimulation of the three different points compared to the baseline resting-state session are depicted in Table 4 and Figure 4A.

**Table 4:** **Brain regions which were detected on the group level in the somatosensory network analysis for the comparison of the post-stimulation resting-state sessions vs. the first resting-state session (baseline), and for the comparison of the three post-stimulation sessions with each other (*P* < 0.05, corrected)**.

**Task**	**Area**	**Left/Right hemisphere**	**Brodmann areas**	**Talairach space, x,y,z**	***T*–value**	***p*–value**	**Volume (mm^3^)**
RS_ST36- RS_B	Anterior cingulate	R	32	6, 34, 22	−7.73	3.59E-07	1728
	Precuneus	R	31	22, −56, 28	7.12	1.17E-06	1377
	Superior frontal gyrus	R	6	8, −16, 65	−7.35	7.48E-07	1215
	Culmen	R	/	28, −47, −18	5.82	1.70E-05	1053
	Parahippocampal gyrus	L	19	−31, −47, −4	6.84	2.06E-06	864
	Fusiform gyrus	L	19	−28, −59, −7	6.72	2.59E-06	783
RS_CP1- RS_B	Medial frontal gyrus	R	6	3, −24, 65	−7.12	1.16E-06	2025
	Culmen	L	/	0, −38, −10	−8.11	1.79E-07	999
	Parahippocampal gyrus	L	19	−31, −62, -7	6.59	3.40E-06	837
	Paracentral Lobule	L	6	−6, −30, 54	−8.11	1.77E-07	783
RS_CP2- RS_B	Parahippocampal gyrus	L	36	−31, −33, −15	6.85	2.01E-06	1053
RS_ST36- RS_CP1	Precuneus/Cuneus	R	7	28, −67, 33	6.51	4.03E-06	2835
	Middle temporal gyrus	R	21	58, −47, 9	7.93	2.50E-07	1674
	Parahippocampal gyrus	R	36	36, −33, −10	7.61	4.54E-07	1242
RS_ST36- RS_CP2	Medial frontal gyrus	L	9	−11, 39, 22	−8.90	4.30E-08	1890
	Inferior frontal gyrus	L	47	−25, 17, −12	−7.58	4.81E-07	1566
	Culmen	R	/	11, −59, −10	6.42	4.82E-06	1053
	Superior temporal gyrus	L	38	−36, 14, −25	−7.01	1.46E-06	891
	Precuneus/Cuneus	R	18	14, −79, 20	6.27	6.51E-06	837
RS_CP1- RS_CP2	Parahippocampal gyrus	R	27	30, −30, −7	−7.79	3.21E-07	2295
	Precuneus	L	19	−34, −73, 38	−8.45	9.57E-08	1161
	Superior temporal gyrus	L	39	−53, −56, 20	−6.03	1.07E-05	918

*Displayed are voxels of maximal significance. If the activated area crosses the midline, only the side of the highest value is displayed. The coordinates are in the Talairach space. T and p value is on the voxel-level*.

ST36 as compared to CP1 revealed a significantly enhanced S2-connectivity to right precuneus, right MTG, and right parahippocampal gyrus (Figure 4B, Table 4).

ST36 as compared to CP2 showed a significantly enhanced S2-connectivity to right precuneus/cuneus and right culmen, whereas the left medial frontal gyrus, left inferior frontal gyrus, and left superior temporal gyrus showed a significantly reduced connectivity to S2 (Figure 4B, Table 4).

Comparing the two control points (CP1-CP2) revealed a significantly reduced S2-connectivity to right parahippocampal gyrus, left precuneus, and left superior temporal gyrus (Figure 4B, Table 4).

The conjunction analyses of the seed-based resting state connectivity for the comparison of acupuncture point vs. control points (conjunction of RS_ST36-RS_CP1 and RS_ST36-RS_CP2), as well as the comparison of two different dermatomes L5 and L2 (conjunction of RS_ST36-RS_CP2 and RS_CP1-RS_CP2) revealed no commonly change in connectivity.

#### Needle sensation

Similar to the EEG experiment the needle sensation was also assessed with the MASS (Kong et al., 2007). The MASS Index for ST36, CP1 and CP2 were 4.71 [3.53;5.89], 3.59 [2.51;4.68], and 3.32 [2.34;4.29], respectively. Differences between ST36 vs. CP1 and ST36 vs. CP2 were statistically significant (pairwise *t*-test, *p* = 0.009 and *p* = 0.005, respectively). There was no significant difference between CP1 and CP2 (*p* = 0.587).

No correlation was found when exploring the relationship between the MASS index and the mean beta values within the ROIs detected in the conjunction analysis (all r-values between −0.48 and 0.51 with *p* > 0.05, corrected for multiple comparison).

## Discussion

We compared the stimulation of the acupuncture point ST36 with two control points that were non-acupuncture points: one near the real acupuncture point in the same dermatome (CP1 in L5) and one in a different dermatome (CP2 in L2). We expected the EEG and fMRI imaging results to be different either when comparing the points in the two different dermatomes (CP2 different from ST36 & CP1), or when comparing the acupuncture point with the two non-acupuncture control points. Comparisons between points in the two different dermatomes (ST36 vs. CP2 and CP1 vs. CP2) showed more pronounced activation at right middle temporal gyrus and deactivation at left superior temporal gyrus when stimulating dermatome L5 (ST36 or CP1). When comparing the acupuncture point with the control points (ST36 vs. CP1 and ST36 vs. CP2) we found (i) pronounced BOLD activation in right insula and right S2, pronounced deactivation in precuneus/PCC, and (ii) a pronounced increase of mu rhythm power in the EEG data following stimulation of ST36. Moreover, increased connectivity of left S2 to the right precuneus was observed in the follow-up resting-state scan for the comparisons of ST36 with the two control points, but in different regions of right precuneus. These results suggest differential processing of acupuncture point stimulation compared to stimulation of non-acupuncture control points, including a potential mechanism of pain modulation due to a complex somatosensory stimulation.

To answer our focused research question we applied a rigorous study design. The subjects were blinded regarding the character of the different point locations and the researchers were blinded during the pre-processing of the data and during the first steps of data interpretation. To prevent systematic errors, different randomization procedures were used. The order of point locations was randomized for both experiments, and the interstimulus intervals were randomized during the fMRI experiment. The washout period of acupuncture stimulation is still unknown. By randomizing the order of point locations all three interventions should be comparably affected by possible carry-over effects. A broad range of somatosensory effects were assessed using EEG, BOLD, and resting-state fcMRI data analysis. Thus, we evaluated event-related changes as well as longer lasting brain activity changes (connectivity and EEG rhythm). Event-related designs can robustly image brain response to discrete, short duration acupuncture stimuli (Napadow et al., 2012) which correspond well with the clinical application of acupuncture stimulation.

Manual acupuncture was chosen because it is more relevant for the clinical setting. But this might also be a cause of systematic error, because acupuncturists obviously could not be blinded in our study. Therefore, we evaluated the needle sensations as reported by the subjects. In part, needle sensations were different between the stimulated points, but we found that sensation was not correlated with brain activity changes. All subjects received the stimulation on all three point locations, therefore the groups we compared were based only on different point locations not on different subjects. We used intra-individual comparisons because the variance of physiological parameters between subjects is typically more pronounced than intra-subject differences caused by an intervention like a somatosensory stimulation on three different points. Because of intra-individual comparisons our data was not independent, though this was taken into account during our statistical analysis. Moreover, age and gender were included into the statistical models as covariates, since these factors might influence the outcome when evaluating the effects of acupuncture. In general, with the subtractive design used in our study, possible interferences between acupuncture effects and the somatotopic organization of evoked brain responses cannot be fully disentangled. This question could be addressed with a 2 × 2 factorial design with two pairs of acupuncture point and control point in different dermatomes, though involving additional experimental effort.

The results of the needle sensation for the two experiments were not comparable. In the EEG experiment the MASS index for ST36 stimulation was similar to CP1 and different from CP2. However, in the fMRI experiment the MASS index for ST36 stimulation was significantly different from the two control points. Several conditions might explain the differences. The position of participants in the two experiments were different. In the EEG experiment, participants were in a sitting position while in a supine position in the fMRI experiment, where the subjects might feel more relaxed than in a sitting position. Moreover, muscle tension on the leg where the needling stimulation was applied can be different in these two positions, and thus influence the sensation processing. In addition, a finer needle (different material and size) had to be used in the fMRI experiment because of the magnetic field of the scanner. Due to the repeated intermittent stimulation in the event-related design of the fMRI experiment, the stimulation protocol might have been more intense than in the EEG experiment.

Many studies show that various forms of somatosensory stimulation (from light touch to painful stimuli) cause transient desynchronization (suppression) of the somatosensory (mu) background rhythm (Neuper et al., 2006; Ploner et al., 2006b; Stancak, 2006). In our study, we observed increased mu rhythm following needle stimulation. Until now, to our best knowledge, such an after-effect of a peripheral somatosensory stimulation has not been described in the literature. A recent EEG study indicated that acupuncture stimulation on acupuncture point LI4 seemed to lead to specific changes in alpha EEG-frequency (Streitberger et al., 2008). However, the authors compared manual penetrating acupuncture with non-penetrating needle stimulation on the same point, i.e., they compared different kinds of stimulation rather than different points. Similar effects of long lasting increased background rhythm have been described for non-invasive brain stimulation protocols such as TDCS/TACS (transcranial direct/alternating current stimulation) or TMS (transcranial magnetic stimulation) (Wagner et al., 2007).

The BOLD activation pattern and connectivity changes we found in our fMRI analysis correspond well with previous findings showing that acupuncture stimulation modulates activity and connectivity of somatosensory as well as pain-related areas (especially insula cortex and S2). Recent neuroimaging studies that compared the stimulation of acupuncture points to control points revealed strengthened BOLD activation in somatosensory areas, the cingulum, the basal ganglia, the brainstem, the cerebellum, as well as the insula cortex. Besides these increases in BOLD activation, these studies also found pronounced acupuncture related deactivation of BOLD signaling in the amygdala, the hippocampus, and brain areas well described as hubs of the brain's default mode network (Dhond et al., 2007; Huang et al., 2012). These observations are in good agreement with the present findings since we also found acupuncture related deactivation in default mode network associated areas and higher BOLD activation in S2 and insula, which are well described as dominant hubs of the central nervous pain network (also known as pain matrix Apkarian et al., 2005; May, 2007). Furthermore, for ST36 as compared to the control points, we found a significantly deactivated right precuneus during stimulation. Although no voxels survived the conjunction analysis for the connectivity comparison of acupuncture point vs. control points, both comparisons (RS_ST36-RS_CP1 and RS_ST36-RS_CP2) showed increased connectivity between right precuneus and S2 in the follow-up resting-state scan. Whereas S2 and insula are assumed to contribute to the experience of pain (Craig, 2009), the precuneus seems to be involved in the assessment and integration of pain (Goffaux et al., 2014). Acupuncture related strengthened functional connectivity between S2 and precuneus might represent a possible mechanism that explains the pain relieving effectiveness of acupuncture, especially in chronic pain (Berman et al., 2010; Vickers et al., 2012). Additionally, our finding of an increased mu rhythm following stimulation of ST36 may represent another potential mechanisms of pain modulation, since an increased mu rhythm was previously shown to be associated with a pronounced cortical inhibition (Klimesch et al., 2007; Jensen and Mazaheri, 2010) and a reduced cortical excitability to painful stimulation (Ploner et al., 2006a). Further studies combining acupuncture with multimodal brain imaging are necessary to test these hypotheses in patients suffering from chronic pain.

Furthermore, we found a pronounced activation at right middle temporal gyrus and deactivation at left superior temporal gyrus during needle stimulation, which was also found by other studies that investigate acupuncture (Zhang et al., 2012; Kim et al., 2013). However, we found these effects only for the comparison between the different dermatomes (L2 and L5) when stimulating dermatome L5 (ST36 or CP1). These effects might hint toward a different sensitivity of the two different regions used for the needle stimulation, but still remain elusive.

In conclusion, our findings suggest that stimulation at acupuncture points may modulate somatosensory and saliency processing regions more readily than stimulation at non-acupuncture point locations. In addition, our results hint toward potential mechanisms of pain modulation due to acupuncture stimulation. Furthermore, our results might have an impact on experiments using conventional somatosensory stimulation protocols. For example, electrical stimulation applied to the median nerve or the finger might produce different imaging results when the stimulation electrodes are located near an acupuncture point. Further experiments using electrical acupuncture and EEG will assess if direct stimulation of acupuncture points also affects established EEG markers, such as evoked potentials or evoked and induced rhythmic activity.

## Funding

This study had no additional funding. Wenjing Huang received a scholarship from the Carstens Foundation. Vitaly Napadow was supported by NCCAM, National Institutes of Health [R01-AT004714, R01-AT005280, P01-AT006663, R21-DK097499, R01-AT007550].

## Author contributions

Conceived and designed the experiments: TN, WH, DP, CMW, AV, VN, FL. Performed the trial: TN, WH, DP. Analyzed the data: TN, XL, SR. Discussed the data: TN, WH, DP, XL, CMW, AV, VN, BP, SR, FL. Wrote the first draft of the paper: TN, DP, WH. Revised the paper and approved the final version: TN, WH, DP, CMW, AV, VN, SR, BP, FL.

### Conflict of interest statement

The authors declare that the research was conducted in the absence of any commercial or financial relationships that could be construed as a potential conflict of interest.
