# Supplementary material for: Differential cerebral response to somatosensory stimulation of an acupuncture point vs. two non-acupuncture points measured with EEG and fMRI
Source: Front Hum Neurosci. 2015 Feb 13;9:74. doi: 10.3389/fnhum.2015.00074 (PMC4327308; doi:10.3389/fnhum.2015.00074)
Supplement: Supplementary file 1 [file Image1.PDF]

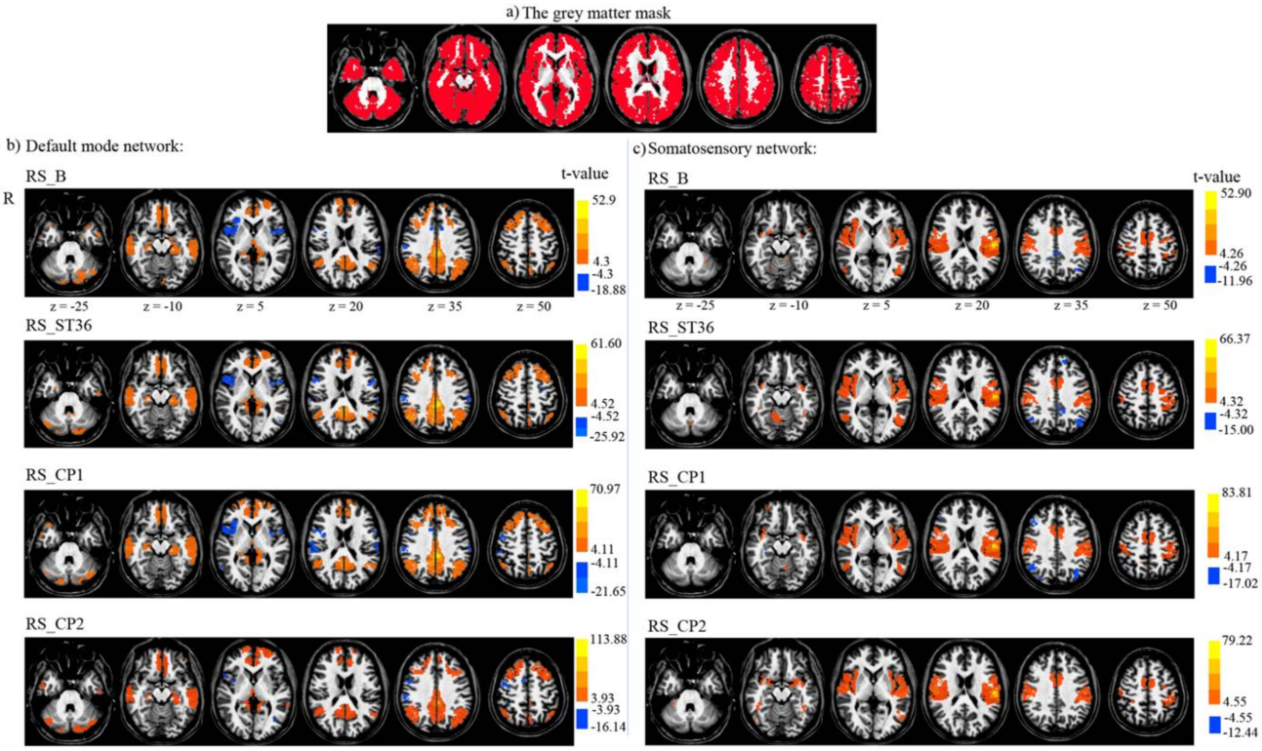

Supplementary figure 1: a) The average grey matter mask derived from the T1 images. b) The default mode network for all resting-state sessions as revealed by seed-based correlation analysis with seed located in posterior cingulate cortex (PCC, Talairach space, x=-2, y=-36, z=37). c) The somatosensory network for all resting-state sessions as revealed by seed-based correlation analysis with seed located in secondary somatosensory cortex (S2, Talairach space, x=-54, y=-21, z=21). R means right hemisphere. Talairach z coordinates are displayed. positive values: red, negative: blue (P<0.05, corrected).
